# Multifaceted Roles of SPL9 in Plant Growth, Development, and Stress Responses: A Review

**DOI:** 10.3390/plants15050814

**Published:** 2026-03-06

**Authors:** Yang Gao, Yu Chen, Jingxia Zhang, Le Zhang, Zhangqiang Song, Furong Wang, Shengli Wang, Jun Zhang

**Affiliations:** 1 Key Laboratory of Cotton Breeding and Cultivation in Huang-Huai-Hai Plain, Ministry of Agriculture and Rual Affairs, Institute of Industrial Crops, Shandong Academy of Agricultural Science, Jinan 250100, China; 2 National Key Laboratory of Cotton Bio-Breeding and Integrated Utilization, School of Life Sciences, Henan University, Kaifeng 475004, China

**Keywords:** transcription factor, *SPL9*, plants developmental progress, biotic and abiotic stress, regulatory network

## Abstract

The *SQUAMOSA PROMOTER BINDING PROTEIN-LIKE* (*SPL*) family, a group of plant-specific transcription factors, plays a key role in plant growth and development through complex regulatory networks. Within this family, *SPL9* has been identified as a key regulator of diverse biological processes. In this review, we summarize the current knowledge on *SPL9*, focusing on its expression regulatory mechanisms and roles in plant development, such as morphogenesis, reproductive processes, and crop yield determinations. We further describe its role in plant responses to abiotic and biotic stresses and its integration into broader regulatory networks. We also outline future research priorities and discuss potential applications of *SPL9*-based strategies in molecular design breeding to increase crop productivity and stress resistance.

## 1. Introduction

Plant development is governed by complex regulatory networks that integrate hormonal, signaling pathways, transcription factors (TFs), and various other molecular regulators. TFs binding to specific DNA sequences can either activate or repress gene expression, modulating diverse plant growth, developmental processes, and stress responses [1,2]. Among the various TF families, the *SQUAMOSA PROMOTER BINDING PROTEIN-LIKE* (SPL) family plays key roles in numerous plant biological processes, including organ development [3,4], plant architecture formation [5], hormone signaling [6,7], and stress responses [8].

The *SPL* genes *AmSBP1* and *AmSBP2* were first identified in *Antirrhinum majus* L., where they were shown to regulate early stages of flower development [9]. After more genomic data can be used, numerous *SPL* genes have been identified in both monocotyledonous [10,11,12,13,14] and dicotyledonous species [15,16,17,18,19,20]. SPL proteins are evolutionarily conserved in land plants and consist of a conserved SBP domain of about 79 amino acids. This domain has two distinct Zn^2+^-binding motifs, Cys-Cys-His-Cys and Cys-Cys-Cys-His, as well as a putative nuclear localization signal [21,22,23]. The SBP domain is necessary and sufficient for binding to the TNCGTACAA sequence in the promoters of target genes, with the GTAC sequence serving as its core motif [23,24].

In *Arabidopsis thaliana*, 17 *AtSPL* genes have been identified, and 10 of which are post-transcriptionally regulated by microRNA156 (miR156) [25,26]. Among them, SPL9 is known to regulate developmental transitions such as the juvenile-to-adult phase change and the onset of reproductive growth [7,26,27]. Subsequent studies have shown that SPL9 plays a critical role throughout almost the entire *Arabidopsis* life cycle, influencing the development of vegetative organs such as lateral roots [28], stem branches [29] and leaves [30], as well as flower meristem activity during the reproductive stage [31,32]. In addition, SPL9 contributes to plant responses to abiotic [33,34] and biotic stresses [35,36], partly through and accompanied by the regulation of the hormone signaling pathway [37].

Notably, studies on *SPL9* have extended beyond *Arabidopsis*. In rice, 19 *OsSPL* genes have been identified and systematically renamed according to their chromosomal positions [38]. Among them, the *OsSPL14*- and *OsSPL17*-encoded proteins cluster within the same phylogenetic clade as AtSPL9 and share significant protein domain similarity, suggesting conserved functions. *OsSPL14*, also known as *IPA1*, (*Ideal Plant Architecture 1*), was independently identified through quantitative trait locus (QTL) analyses: one focused on plant architecture [39], and another linked the *WFP* (*WEALTHY FARMER’s PANICLE*) locus to primary panicle branching [40]. It has been confirmed that *IPA1* enhances rice plant architecture by reducing tillering and improving yield, while also increasing disease resistance [41,42,43]. Its broad impact has led to the recognition of *IPA1* as a potential new “Green Revolution” gene for rice improvement [44].

Due to its pleiotropic roles across tissues and developmental stages, hormone signaling, and responsiveness to environmental stresses, SPL9 represents a key regulatory node in plant biology. Research on SPL9 not only deepens our understanding of fundamental plant developmental mechanisms but also provides valuable insights for crop improvement. In this text, we summarize the current literature on expression regulation, biological function and stress role of *SPL9* as well as its regulatory networks. And we discuss the potential applications of SPL9 in future crop breeding programs.

## 2. Coordinated Multilayer Regulation of SPL9 Expression and Activity

**Figure 1:**
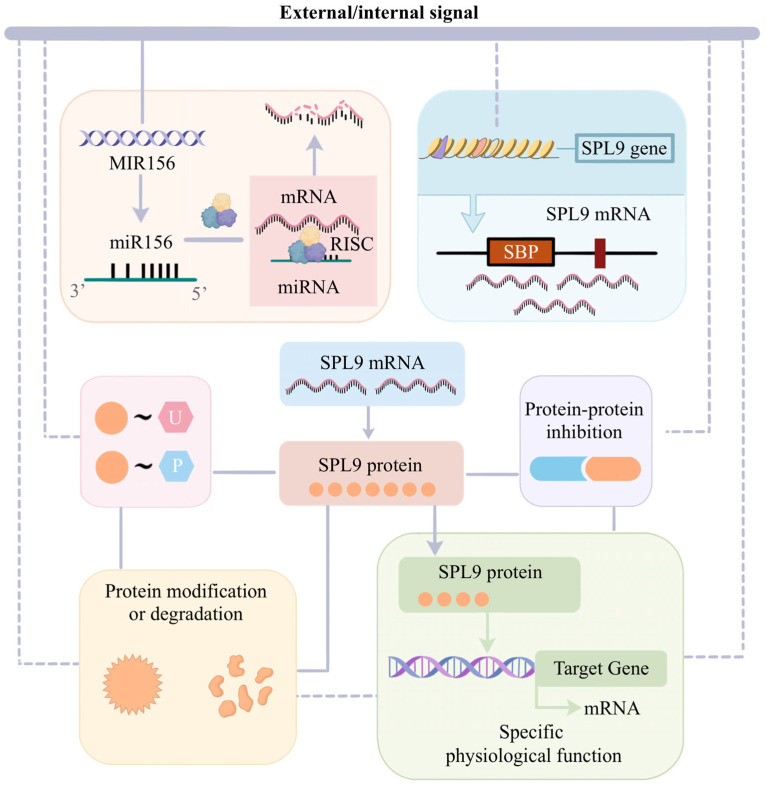
A multi-tiered regulatory network of SPL9. This model illustrates the coordinated integration of three distinct regulatory layers: transcriptional control, post-transcriptional fine-tuning, and post-translational modification, which collectively ensure precise spatiotemporal control of SPL9 activity.

As a key transcription factor involved in plant growth and development, *SPL9* is expressed in multiple tissues, including roots, stems, leaves, and floral organs. Based on the AtGenExpress expression data, *AtSPL9* exhibits high expression in the shoot apex during inflorescence and transition, as well as in early floral organs and carpels; moderate expression is observed throughout the rosette following the transition to flowering; it is also expressed in the seedling hypocotyl, roots and various other organs [45]. The functional output of SPL9 is governed by a sophisticated, multi-layered regulatory mechanism. It integrates transcriptional control, post-transcriptional fine-tuning by miR156, and post-translational regulation of protein activity and stability. In detail, transcriptional regulation defines the potential for *SPL9* production, miR156-mediated post-transcriptional regulation quantitatively limits the translatable mRNA pool, and post-translational mechanisms act as a final functional valve, determining the activity, specificity, and stability of the SPL9 protein (Figure 1).

### 2.1. Transcriptional Regulation of SPL9 Expression

Regulations at the transcriptional level of SPL9 are mediated by specific transcription factors and epigenetic mechanisms at key developmental transitions. These regulated changes in *SPL9* transcriptional levels establish the foundation for any subsequent downstream regulation. For instance, during the vegetative phase transition, MYB33 directly binds to a specific region (−585 to −690 bp) of the *AtSPL9* promoter and enhances its transcription [46]. This transcriptional upregulation increases the substrate mRNA pool that is subject to subsequent fine-tuning by miR156 at the post-transcriptional level. During sucrose-induced juvenile-to-adult transition, PAP1 activates *AtSPL9* expression by directly binding to the MYB-binding motif (MYB-BM) in its promoter sequence in *Arabidopsis*, which occurs in conjunction with glucose-mediated repression of *MIR156* [47].

In rice, a 54 bp deletion in the promoter of *IPA1* (a rice ortholog of *AtSPL9*) leads to increased transcriptional activity and improved agronomic traits, including increased panicle number and size [48]. This deletion removes An-1 binding to the GCGCGTGT cis-element and frees *IPA1* expression from An-1 mediated repression, which increases the functional protein that regulates downstream target genes and thus improves key traits [48]. Whereas in alleles with an intact An-1 binding site, *IPA1* transcription is suppressed. This results in robust vegetative growth similar to high miR156 conditions [39]. In addition, epigenetic mechanisms also influence the transcriptional control of *SPL9*. For example, high expression of *IPA1* is associated with DNA hypomethylation at GHH-type cytosine sites within the 800 bp genomic region upstream of the ATG start codon in rice [42]. This indicates that stable epigenetic modifications can influence the basal transcription level of SPL9 orthologs.

### 2.2. Post-Transcriptional Regulation of SPL9 by miR156

Based on transcriptional control, the conserved miR156 determines SPL9 protein accumulation by targeting and degrading SPL9 mRNA. *SPL9* mRNA contains the miR156 response element in the 3′ untranslated region (UTR), making it susceptible to miR156-guided RNA-induced silencing complex (RISC) [8,25]. Base-pairing with its target mRNA guides the RNA-induced silencing complex (RISC) to direct AGO protein-mediated transcript cleavage and degradation [49]. Even without complete degradation, the RISC bound to *SPL9* transcripts blocks their translation, forming a translational barrier to limit the functional SPL9 protein production. It thus provides an important upper limit on SPL9 protein accumulation.

The expression of *MIR156* genes is also precisely controlled in order to provide the signal integration node. The abundance of miR156 is dynamically regulated by a series of internal and external cues, which in turn fine-tune the miR156-mediated post-transcriptional repression of *SPL9* across diverse developmental programs and stress responses [50]. In the context of the vegetative phase change, miR156 is not only regulated by developmental age but is also profoundly influenced by metabolic signals. For instance, glucose represses miR156 transcription and facilitates the degradation of its primary transcripts through a pathway involving Hexokinase 1 (HXK1) [47]. The age-dependent decline in miR156 is also reinforced by epigenetic modifications. Complexes such as PRC1, PRC2 and the PKL-HDA9 chromatin remodeling machinery establish a repressive chromatin state at specific loci such as *MIR156A* and *MIR156C*, thereby ensuring the irreversibility of the transition from juvenile to adult phase [51].

Precise regulation by miR156 fine-tunes the abundance of a suite of downstream SPL transcription factors. In *Arabidopsis*, at least ten *SPL* genes are validated targets of miR156, which can be functionally classified according to their roles in different developmental stages. For example, SPL9, SPL10, SPL13 and SPL15 regulate the vegetative phase change. Although functional redundancy exists among these members, SPL9 exhibits a uniquely broad and central role. It expands its functional scope by directly targeting and regulating key genes responsible for adult traits. During leaf development, SPL9 directly binds to the promoters of leaf morphology regulators such as *BOP1*, *BOP2*, and *CYCD3* to control their expression [30]. While the SPL family collectively serves as an integrator of flowering signals, SPL9 adopts a distinct strategy compared to other members such as SPL3, SPL4, and SPL5, which directly activate flowering integrator genes [31], and functions as a developmental timer by activating the expression of *miR172*, which regulates downstream targets to flower [52]. Similarly, in the control of axillary bud outgrowth and branching, although SPL9 and SPL15 show redundant functions in certain light signaling pathways, only SPL9 has been demonstrated to uniquely integrate the miR156 module with the gibberellic acid (GA) signaling pathway to fine-tune branching architecture [29,37,53]. Collectively, these findings position *SPL9* as a leader among the miR156-targeted *SPL*s, playing an indispensable and overarching role in multiple core developmental processes. Subsequent chapters will address how SPL9 regulates its target genes and the physiological mechanisms in plant development.

### 2.3. Protein Interaction and Post-Translational Modifications

The functional output of SPL9 is ultimately regulated by protein–protein interactions and post-translational modifications (PTMs). SPL9 transcriptional activity can be suppressed through direct or indirect suppression of protein–protein interactions, which maintain SPL9 in an inactive state until appropriate signals are perceived. DELLA proteins, plant-specific regulatory proteins lacking a DNA-binding domain, act as growth repressors by interacting with other TFs, including SPL9 [54,55,56]. They are usually bound to the C-terminal region of AtSPL9, which inhibits its ability to activate downstream genes in response to gibberellin signaling [57]. The critical link to upstream regulation is that GA signaling triggers DELLA degradation, thereby releasing the pre-existing suppressed SPL9 protein [37]. Other repressors such as DEWAX and strigolactone signaling components D53/SMXLs directly interact with SPL9 in the form of non-functional complexes, storing the TF and preventing target gene activation [29,58].

Post-translational modifications (phosphorylation and ubiquitination) provide a rapid and reversible means to fine-tune the activity, stability, and functional specificity of the SPL9 protein pool. Phosphorylation can inhibit or redirect SPL9 function. For example, BIN2 kinase phosphorylates AtSPL9 to suppress its role in the vegetative phase transition [7]. Pathogen-induced phosphorylation of IPA1 at Ser163 alters its DNA-binding specificity, enabling it to activate the immunity gene *WRKY45* instead of its growth targets [59]. Ubiquitination also regulates SPL9 abundance, which is essential for maintaining a balance between growth and stress resistance [60,61,62]. The RING-finger E3 ligase IPA1 INTERACTING PROTEIN1 (IPI1) mediates the tissue-specific ubiquitination of IPA1 by means of different polyubiquitin chains, thereby modulating IPA1 complex stability and maintaining appropriate protein levels across tissues [62].

## 3. The Diverse Role of SPL9 in Plant Growth and Development

Plant growth and development are highly ordered and regulated processes involving numerous regulatory mechanisms and complex signaling networks. Among the main regulators, *SPL9*, a plant-specific TF targeted by miR156, forms a core regulatory module that integrates diverse internal and external cues to coordinate various developmental programs. SPL9 functions in each of these developmental pathways are determined by multilevel regulatory mechanisms that limit its expression and activity. In this section, we highlight the indispensable roles of SPL9 across multiple stages of development covering both below-ground and above-ground traits. Above-ground traits are subdivided into juvenile-to-adult phase change, shoot branching and tillering, and age-dependent flowering. Table 1 summarizes the main mechanisms for SPL9 activity, along with its key targets and their functions across these physiological processes.

### 3.1. SPL9 Regulates Lateral Root Development and Nodulation

**Figure 2:**
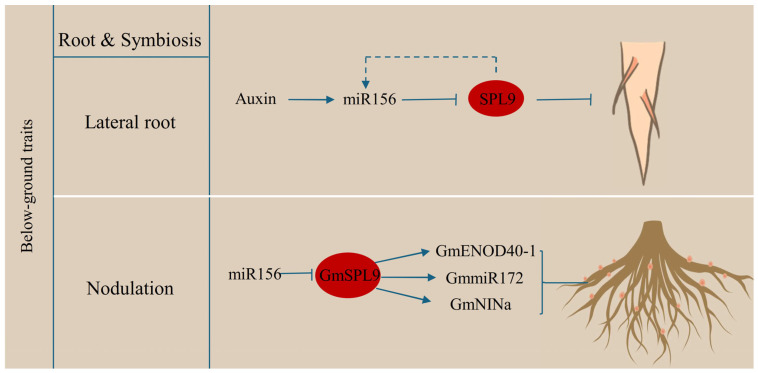
Diagram of SPL9 involvement in below-ground traits. SPL9 plays a regulatory role across multiple stages of plant development. In root and symbiosis, as a core component of the miR156/SPLs module, SPL9 participates in lateral root primordia progression by responding to auxin signals in *Arabidopsis*. Another, in soybean, GmSPL9 enhances nodulation by promoting *miR172*, *GmNINa* and GmENOD40-1 expression. Arrows represent activation, T-bars represent inhibition.

The root system is essential for absorbing water and nutrients as well as for sensing changes in the soil environment. Lateral roots, as an important component of root architecture, originate from founder cells within the pericycle, located opposite the xylem poles. Following several rounds of division, these cells give rise to lateral root primordia from the primary root [28,69,70], which are regulated by hormones, miRNAs, and TFs [64,70,71,72]. The conserved miR156-SPL module acts antagonistically, in which miR156 promotes lateral root formation, while *SPL9* and its homologs suppress it [63]. Both miR156 and its targets (*SPL9* and *SPL10*) are responsive to auxin signals, with *SPL9*/*SPL10* mediating mir156 levels to create feedback loops that stabilize lateral root development and integrate auxin into miR156/SPL module in *Arabidopsis* (Figure 2). The evolutionary conservation of SPL9’s role in lateral root development is illustrated by the influence of the miR156-StSPL9 module on both plant height and root architecture in potato [71].

In addition to developmental regulation, SPL9 mediates environmental responses in roots, such as symbiotic nodulation in legumes under low-nitrogen conditions or rhizobial infection (Figure 2). In soybean, the miR156b-GmSPL9d module is involved in nodulation. *GmSPL9* is downstream of rhizobia-suppressed miR156, leading to increased protein abundance. GmSPL9d binds to the promoters of *miR172* and nodulation markers such as *GmNINa* and *GmENOD40-1*, thereby integrating nodulation signaling with root development [64]. However, excessive nodulation is harmful because of the maintenance of nodules consumes considerable energy. In *Medicago sativa* (alfalfa), *MsSPL9* suppresses nodulation in response to nitrate signals. Silencing *MsSPL9* can alleviate nitrate stress and disrupt the expression of nitrate-responsive and nodulation genes [73].

### 3.2. SPL9 Modulates Vegetative Phase Change

**Figure 3:**
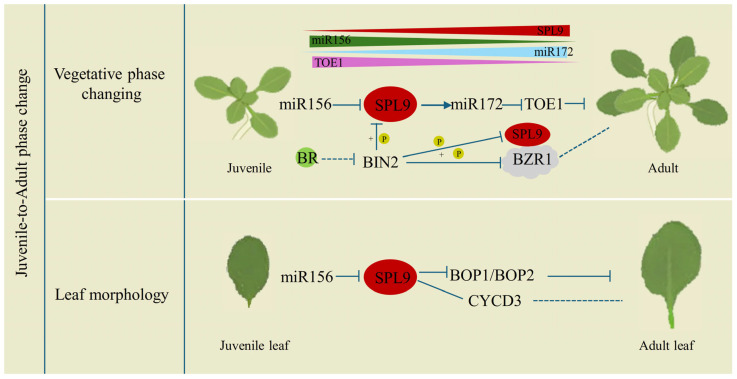
Diagram of SPL9 involvement in juvenile-to-adult phase change in *Arabidopsis*. In vegetative phase change, SPL9 integrates both the age pathway and the brassinosteroid (BR) signaling pathway. Declining miR156 levels lead to increased *SPL9* expression, which in turn upregulates miR172 expression, repressing its target *TOE1*. In leaf morphogenesis, SPL9-BOP1/BOP2 module regulates the proximal-distal leaf patterning. Meanwhile, the SPL9-CYCD3 module coordinates cell proliferation with development timing, ensuring age-dependent organ morphology. Arrows represent activation, T-bars represent inhibition.

Postembryonic ontogeny is characterized by distinct phase transitions, with the juvenile-to-adult vegetative shift being crucial for growth and reproduction. miR156 is a key regulator of this process, and its target gene, *SPL9*, plays a crucial role [26,74]. The level of miR156 is high in the juvenile phase and decreases with increasing age, while the expression of *SPL9* increases. AtSPL9 promotes the transcription of *miR172*b and accelerates the transition to the adult phase [65]. A nonsynonymous mutation in the nuclear localization signal (NLS) of the SBP domain in SPL9 delays the vegetative phase change in *Arabidopsis* [75]. Similarly, the loss-of-function mutation *dwf5* results in a delayed phase transition due to decreased *AtSPL9* and *miR172* levels and increased accumulation of TARGET OF EAT1 (TOE1) [7]. BIN2, a GSK3-like kinase that negatively regulates BR signaling [76,77,78], interacts with and phosphorylates both AtSPL9 and TOE1, promoting their degradation. Reduced BR levels increase BIN2 activity, destabilize AtSPL9 and TOE1, downregulate miR172, and delay phase transition [7]. SPL9 functions as a central integrator within the miR156-SPL9-miR172-TOE1 module, linking BR signaling to developmental timing (Figure 3).

The transition to adult vegetative growth in plants is often accompanied by heteroblastic changes in leaf morphology and arrangement [79,80]. *SPL9* plays a key role in this process [26,81]. Together with its homolog *AtSPL13*, *AtSPL9* promotes leaf blade elongation, resulting in larger, more elongated leaves [30]. They bind directly to the promoter regions of *BOP1* and *BOP2* to suppress their expression, which prolongs blade development while retarding petiole development in *Arabidopsis* [30] (Figure 3). Although AtSPL9 and AtSPL13 share certain binding sites within the genomic regions of *BOP1* and *BOP2*, they also display distinct binding profiles, which may account for the shared and unique phenotypic traits of *rSPL9* and *rSPL13* in *Arabidopsis*.

Notably, *AtSPL9* is absent in juvenile leaves, which have small and round blades distinctly separated from the basal petiole. However, *SPL9* is expressed in adult leaves, which are larger, oval-shaped, and feature a more gradual transition between the blade and petiole in *Arabidopsis* [27]. Cytologically, AtSPL9 prolongs leaf morphogenesis by maintaining cell proliferation and delaying the transition to endoreduplication and differentiation [27]. AtSPL9 directly targets *CYCD3* genes to regulate the cell cycle, thus influencing leaf heteroblasty [27] (Figure 3).

### 3.3. SPL9 Controls Branching and Tillering

**Figure 4:**
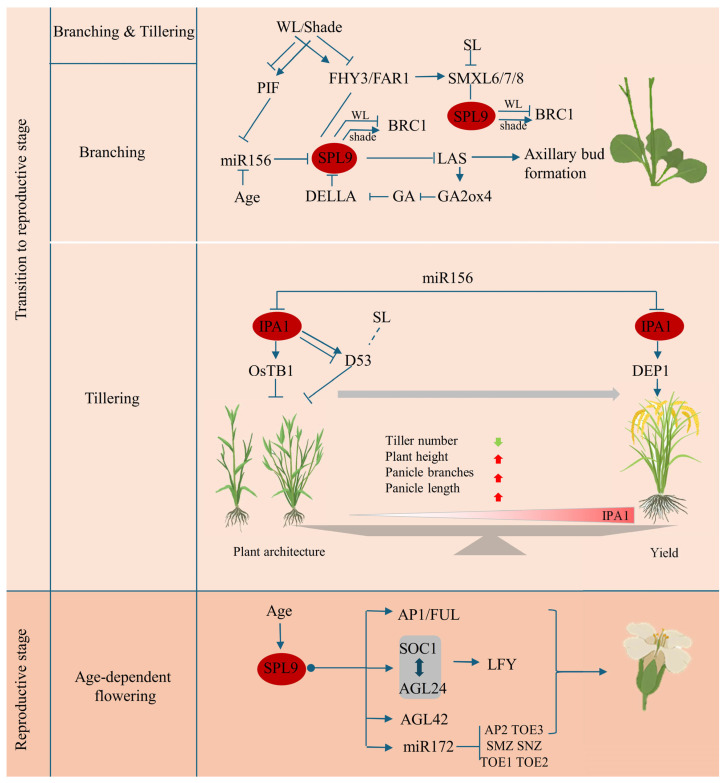
Diagram of SPL9 involvement in late vegetative development and flowering regulation. SPL9 participates in the regulation of axillary bud development through gibberellin signaling pathway, the age-development pathway, and the integration of light and strigolactone signaling pathways in *Arabidopsis*. SPL9 binds to and represses LAS transcription, thereby affecting the axillary bud formation. The SPL9-LAS-GA pathway forms a feedback loop. DELLA proteins interact with SPL9, inhibiting its activity and easing repression on LAS, thus promoting axillary bud development. Conversely, LAS enhances GA2ox4 expression, reducing GA levels and increasing DELLA accumulation. Under white light (WL), FHY3/FAR1 and SMXL6/SMXL7/SMXL8 interact with SPL9 and suppress its transcriptional activation of BRC1, promoting branching. In shaded conditions, reduced FHY3/FAR1 and SMXL levels alleviate SPL9 inhibition, increasing *BRC1* expression and suppressing branching. SMXL proteins are involved in strigolactone-induced degradation of D53-like proteins, influencing branching. In the process of shaping rice architecture, *IPA1* controls tillering through upregulation of *OsTB1* and involvement in the strigolactone pathway. At the maturity stage, increased expression of IPA1 promotes the expression of the yield-enhanced factor DEP1, improving yield traits like plant height, panicle branches and panicle length. During reproductive development, SPL9 promotes flowering in *Arabidopsis* via the age-dependent pathway, directly activating flowering regulators such as *AP1*, *FUL*, *SOC1*, *AGL24* and *LFY*. Arrows represent activation, T-bars represent inhibition.

SPL9 plays a significant role in regulating the development of lateral organs, such as branches in *Arabidopsis* and tillers in rice (Figure 4). In *Arabidopsis*, SPL9 fine-tunes branching by integrating age, hormone, and light signals. Under shade conditions, the accumulation of phytochrome-interacting factors (PIFs) directly binds to the *MIR156* promoter to suppress its expression [53]. This suppression reduces the post-transcriptional regulation of miR156 on its target genes. Light and hormone signaling converge on AtSPL9 and AtSPL15, with both light-sensitive factors FHY3/FAR1 and strigolactone-associated SMXL6/7/8 physically interacting to inhibit their transcriptional activation of BRC1 [29]. Reduced FHY3 and FAR1 release SPL9/SPL15 from repression by FHY3/FAR1 and SMXL6/7/8, enabling them to activate BRC1 expression and suppress branching. Furthermore, PIF4 physically interacts with AtSPL9 to activate *BRC1* and modulate branching [82]. Beyond its overlapping function with SPL15, SPL9 plays a distinct role in modulating axillary bud formation. It directly represses LATERAL SUPPRESSOR (LAS) by binding to its promoter, thereby inhibiting axillary bud formation [37]. This repression is counteracted by regulatory inputs from the gibberellin signaling pathway. DELLA proteins, key components of gibberellin signaling, interact with AtSPL9, diminishing its repressive effects on LAS and promoting axillary bud formation. This process operates through a GA-responsive feedback loop involving the DELLA-SPL9-LAS-GA2ox4 regulatory module [37].

*IPA1* in rice regulates tillering and yield, primarily through its involvement in the strigolactone (SL) signaling pathway. In this pathway, IPA1 and D53 (a known SL signaling repressor) form a feedback loop [58,83]. By binding to the *D53* promoter, IPA1 activates its expression, while the D53 protein interacts with IPA1 to inhibit its transcriptional activity. This interaction is modulated by SL perception, which induces D53 degradation and consequently releases IPA1 to suppress tillering [58]. Additionally, IPA1 directly activates *OsTB1*, a gene from the TCP (TEOSINTE BRANCHED1/CYCLOIDEA/PROLIFERATING CELL FACTOR) family that inhibits axillary bud outgrowth, and binds to the promoter of *DEP1* (DENSE AND ERECT PANICLE 1), influencing both panicle architecture and grain yield [41,66]. Thus, *IPA1* reduces tillering, increases plant height, and enhances panicle branching, contributing to an ideal plant architecture. Moreover, IPA1 recognizes novel cis-elements, such as the TGGGCC/T motif, through its interaction with PCF1 and PCF2, thereby expanding its regulatory targets [41].

### 3.4. SPL9 Roles in the Age-Dependent Flowering Pathway

The age-dependent flowering pathway relies primarily on intrinsic developmental signals, rather than external environmental cues [31,84]. The miR156-targeted *SPL* gene module regulates the vegetative-to-reproductive transition and flowering time by coordinating the expression of flowering-related genes across the age gradient [31,74,84,85]. As a key target of miR156, *SPL9* has highly conserved flowering regulatory functions across multiple species [85,86,87]. In *Arabidopsis*, the levels of *AtSPL9* and *AtSPL3* in the shoot apices increase with age. The overexpression of these genes accelerates flowering, whereas elevated miR156 delays flowering by suppressing SPL activity [74,81,88]. Increased SPL9 levels promote flowering by directly binding to the GTAC core motif in the regulatory regions of positive floral regulators, including *FUL*, *SOC1*, *AGL24* and *AGL42*, activating their expression [31,32]. Concurrently, SPL9 acts upstream of miR172, directly promoting its transcription. The upregulation of miR172 establishes a crucial indirect route for SPL9 to regulate flowering. miR172 in turn post-transcriptionally represses a suite of its own target genes, which encode AP2-like transcriptional repressors of flowering (e.g., TOE1, TOE2, TOE3, SMZ, SNZ) [52]. The suppression of these repressors further facilitates the floral transition. Thus, through this coordinated miR156-SPL9-miR172 regulatory module, SPL9 integrates direct gene activation with indirect de-repression to ensure a timely and precise onset of flowering (Figure 4).

### 3.5. Additional Developmental Functions of SPL9

In addition to its well-established roles in vegetative and reproductive development, SPL9 plays critical regulatory roles in seemingly disparate processes such as trichome initiation and cuticular wax biosynthesis.

In *Arabidopsis*, trichome initiation and development are regulated by positive regulators (e.g., TTG1, GL1, GL3, EGL3) [89,90,91,92] and by negative regulators from the R3 MYB factors (e.g., TRY, TCL1/2) [93,94,95,96,97,98]. AtSPL9 suppresses trichome initiation by activating the expression of *TRY* and *TCL1* [99]. This pathway functions independently of the GIS-dependent trichome pathway [100,101]. LOM (LOST MERISTEMS) proteins (LOM1/2/3), which are targeted by timing miR171, interact with AtSPL9 to promote trichome formation and delay flowering [67]. In addition, TOE1, a downstream target of the miR156-SPL9 module, represses GL1 expression, thereby inhibiting trichome development [50].

SPL9 also promotes cuticular wax deposition in *Arabidopsis* via the DEWAX/SPL9-CER1 [68,102]. *AtSPL9* expression follows a circadian rhythm, peaking during the light period and declining in darkness [68]. Under light, SPL9 directly influences the expression of wax biosynthesis genes, such as *ECERIFERUM1* (*CER1*), and indirectly affects *CER4* [68]. At night, DEWAX protein accumulates and directly interacts with SPL9, inhibiting its DNA-binding activity and suppressing wax synthesis [68]. Two MYB-SHAQKYF TFs, MYS1 and MYS2, function upstream of the DEWAX-SPL9 module, repressing DEWAX expression while promoting CER1 expression [102]. Although SPL9 and SPL13 play redundant roles in regulating wax biosynthesis under drought conditions, SPL13 is not involved in the DEWAX-mediated diurnal control of wax production [34].

## 4. The Multifaceted Regulatory Roles of SPL9 in Plant Stress Responses

Plants are constantly exposed to a wide array of environmental stresses that threaten their growth, development, and survival. Recent studies have suggested that SPL9 is a multifunctional modulator, coordinating plant responses to both biotic and abiotic stresses. Central to this regulation is the involvement of the miR156-SPL9 module, which integrates pathways related to secondary metabolite production and phytohormone signaling (see Table 2). The following sections summarize the roles of SPL9 in mitigating abiotic stresses, such as drought and temperature extremes, as well as biotic stresses, including pathogen infections and insect herbivory. We focus on the dual regulatory function of SPL9 as a transcriptional activator and repressor to fine-tune defense responses while balancing the growth-defense trade-offs.

### 4.1. Drought Stress

**Figure 5:**
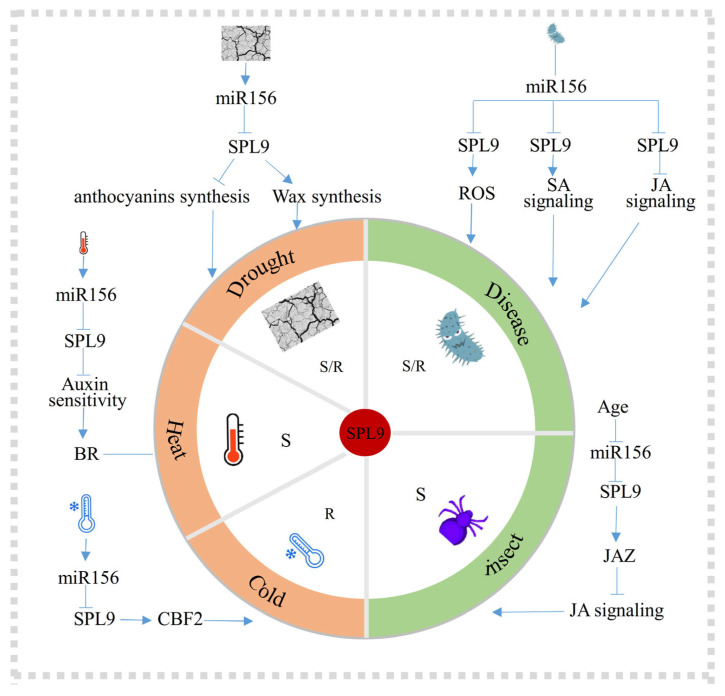
Overview of SPL9’s involvement in stress response pathways in plants. SPL9 participates in various abiotic and biotic stress responses in *Arabidopsis*, such as drought stress, heat response, cold resistance, viral and pest defense. Under drought stress, SPL9 promotes wax biosynthesis to enhance drought resistance, whereas inhibiting anthocyanin synthesis, thereby sensitizing the plant to drought stress. In response to heat stress, SPL9 is involved in a mir156-dependent hormonal pathway to manage temperature change. Under cold stress, SPL9 can upregulate the expression of the cold-responsive transcription factor *CBF2* and increase plant cold resistance. In virus infection, SPL9 promotes ROS accumulation and positively regulates salicylic acid (SA) signaling, increasing the plant’s innate immunity. Conversely, SPL9 inhibits the expression of genes in the jasmonic acid signaling pathway, weakening resistance of plants against viruses. In the same way, *SPL9* negatively regulates plant defense against insects. Saffron yellow areas represent abiotic stress, and green areas represent biotic stress. “S” stands for sensitivity, “R” stands for resistance. Arrows represent activation, T-bars represent inhibition, and lines represent association.

In response to drought stress, SPL9 negatively modulates antioxidant defense mechanisms and positively stimulates cuticular wax biosynthesis (Figure 5). One of its key functions is regulating anthocyanin synthesis in response to drought stress [103,106,107]. Anthocyanins are strong antioxidants, and their synthesis follows a pathway with the enzyme DIHYDROFLAVONOL-4-REDUCTASE (DFR) as the core. The expression of *DFR* is controlled by the MYB-bHLH-WD40 complex [103]. AtSPL9 interacts with PAP1, a core MYB component of the MYB–bHLH–WD40 complex, and negatively regulates *DFR* expression, thereby suppressing anthocyanin synthesis. This competitive interaction prevents PAP1 binding to TT8 (a bHLH factor), thereby destabilizing the transcriptional activation complex and reducing DFR expression. Thus, SPL9 fine-tunes anthocyanin synthesis, resulting in the negative regulation of drought stress responses. The contribution of SPL9 to drought tolerance through its antioxidant defense mechanism has been evaluated in some species. In cassava, *MeSPL9-RNAi* plants have high anthocyanin levels with improved drought resistance [108]. In rice, IPA1 positively regulates drought tolerance by activating the expression of *SNAC1* [109], an NAC TF that plays a key role in reactive oxygen species (ROS) homeostasis [110]. This mechanism enhances drought resistance by promoting ROS scavenging.

SPL9 also acts as a positive regulator of cuticular wax deposition, which forms a critical barrier against non-stomatal water loss. Here, SPL9 functions downstream in the MYS1/MYS2-DEWAX-SPL9 module. The upstream regulators MYS1 and MYS2 are likely responsible for activating or stabilizing SPL9, which in turn promotes the expression of wax biosynthesis genes [102] (Figure 5). Overexpression of *AtSPL9* rescues the drought hypersensitivity of *mys1c mys2* mutants. Both *AtSPL9* and *AtSPL13* are involved in cuticular wax biosynthesis under drought stress [34]. Plants overexpressing *AtSPL9* or *AtSPL13* presented reduced transpiration rates, whereas single and double mutants showed increased water loss.

### 4.2. Temperature Stress

Extreme temperatures, both heat and cold, are major stressors that impact plant growth and development. miR156 expression is induced by high temperature and serves as a key regulator by targeting SPLs to mediate thermal responses. Among these targets, SPL9 has also been identified as a key member that mediates thermal responses [111,112] (Figure 5). The essential role of miR156 in thermomorphogenesis is demonstrated by the reduced hypocotyl elongation observed in miR156-knockdown (*MiM156*) plants [104]. Conversely, elevated *AtSPL9* inhibits hypocotyl elongation in both *MiM156* and *rSPL9*-overexpressing lines. The miR156-SPL9 module acts antagonistically to regulate hypocotyl elongation during thermomorphogenesis, enabling plants to adapt their growth patterns through the modulation of auxin sensitivity in *Arabidopsis* [104].

SPL9 also plays a vital role in cold stress responses (Figure 5). SPL9 expression is induced by low temperatures across various species [33,113]. Despite a concurrent increase in *miR156* expression under cold stress, the induction of *SPL9* appears to operate through a pathway that is independent of miR156-mediated targeting [33]. SPL9 cooperates with other TFs to enhance cold tolerance. Notably, SPL9 may bind to the promoter of *CBF2*, a member of the C-REPEAT-BINDING FACTOR/DRE-BINDING FACTOR1 (CBF/DREB1) family, to activate its expression [33,114]. In rice, the OsSAPK6-IPA1-OsCBF cascade is activated in response to cold stress [105]. IPA1 is phosphorylated by OsSAPK6 at Ser213, which stabilizes IPA1 and promotes cold adaptation. Furthermore, IPA1 activates *OsCBF3* expression by binding to the GTAC core motif within its promoter, confirming that SPL9 is a regulator of cold tolerance.

### 4.3. Biotic Stress

SPL9 is also implicated in plant immunity against pathogens and insects (Figure 5). For instance, *AtSPL9* promotes ROS accumulation and increases the expression of SA-responsive genes, including SA-induced pathogenesis-related (PR) genes *PR1* and *PR2* [35]. The miR156-SPL9 module further regulates phytohormone-responsive genes to trigger innate immunity [35,36]. Salicylic acid (SA) functions as a central defense hormone that activates immune responses against bacterial pathogens [115]. *Arabidopsis* plants in which *miR156* was suppressed or *SPL9* was overexpressed showed milder symptoms upon infection with *Psedomonas. syringae* pv. *tomato DC3000*, along with the upregulation of SA-dependent defense genes [35]. The role of SPL9 in biotic stress responses is notably context-dependent, varying with the type of pathogen involved [35,36]. During infection by the necrotrophic fungus *Botrytis cinerea*, the miR156-SPL9 module links the function of SQUINT (SQU), which is an ortholog of the immunophilin cyclophilin 40 (Cyp40), with jasmonate (JA) signaling in *Arabidopsis* [36]. SQU promotes miR156 accumulation to repress SPL9, thereby relieving the SPL9-mediated suppression of JA signaling [36].

Under insect attack, SPL9 functions as a negative regulator of JA-mediated defense responses [36,108]. *AtSPL9* directly suppresses the JA signaling pathway, diminishing the plant’s resistance to insect attack [6]. Experimental evidence demonstrates that overexpression of miR156 represses SPL9, leading to the elevated expression of JA-responsive genes such as *LOX2* and *VSP2* [6]. These findings further underscore SPL9’s role as a suppressor of JA-dependent defense. Specifically, SPL9 interacts with JA ZIM-domain (JAZ) proteins, promoting and stabilizing their accumulation, which represses JA signaling and weakens JA-dependent insect resistance [6].

## 5. Conclusions and Perspectives

SPL9 is a key transcription factor involved in regulating plant growth, developmental phase transitions, and biotic and abiotic stress responses. Its expression is tightly controlled by miR156, forming a conserved miR156-SPL9 regulatory module across multiple crop species [39,64,71]. SPL9 acts as a transcriptional regulatory hub in complex networks, interacting with multiple genes and pathways. It primarily modulates downstream targets by binding to the GTAC core motif and/or TGGGCC/T motif, as demonstrated in the regulation of age-dependent flowering [31,32]. The transcriptional activity of *SPL9* is dynamically influenced by developmental timing, environmental cues, and hormone signaling. For instance, during inflorescence stem branching in *Arabidopsis*, *AtSPL9* expression is modulated by age-dependent pathways, light conditions, and hormonal inputs, highlighting its integrative role in multiple regulatory signals [29,37]. However, the molecular mechanisms involved in cross-talk between SPL9 and these pathways remain poorly understood.

We summarize the existing knowledge on SPL9 in terms of vegetative growth, reproductive development, transitions of developmental stages, and stress responses. By fine-tuning its spatiotemporal expression, SPL9 selectively regulates downstream genes to direct organ formation at precise stages and locations, a mechanism critical for achieving optimal plant architecture. SPL9 also serves as a mediator and regulator of stress responses by modulating secondary metabolic and phytohormone pathways. Currently, it is still not fully understood how SPL9 might serve as a convergence point for developmental and environmental signals to regulate the trade-off between growth and defense.

Research on *SPL9* has been extensively conducted in model species such as *Arabidopsis thaliana* and rice, but the orthologous genes in other crops are largely unexplored (Table S1). Future research should focus on elucidating the conserved and divergent functions of *SPL9* orthologous genes, as well as revealing the molecular mechanisms that govern their spatiotemporal expression and integration of developmental and environmental signals. Simultaneously, modern biotechnological tools such as CRISPR-based genome editing, high-throughput phenotyping, and systems-level omics are used to target the regulation of SPL9 and analyze regulatory networks, and there is a potential to optimize agronomic traits in molecular design breeding. These efforts lay the foundation for the regulatory networks mediated by SPL9 and also create practical strategies for improving agricultural productivity and enhancing stress resistance.

## Figures and Tables

**Table 1:** Overview of SPL9 and its regulatory role in plant growth and development.

Process	SPL9	Activity Regulation	Key Targets	Primary Function
Later root development	*AtSPL9*	miRNA-target	NA	mediate lateral root development in *Arabidopsis* [63]
Nodulation	*GmSPL9*	miRNA-target	GmNINa, GmENOD40-1, GmmiR172	Act as an upstream master of soybean nodulation [64]
Vegetative phase change	*AtSPL9*	miRNA-target; fine-tuned by protein modifications	miR172	Orchestrates the adult phase transition [7,65]
Leaf development	*AtSPL9*	miRNA-target	BOP1, BOP2, CYCD3	Play a role in leaf morphology and arrangement [27,30]
Branching	*AtSPL9*	miRNA-target and DELLA	BRC1	Integrates internal and external signals to coordinately control branching [29,37]
Tillering	*IPA1*	miRNA-target; Protein–protein interaction	OsTB1, OsPCF1, OsPCF2	Balancing tillering and yield [41,58,66]
Flowering	*AtSPL9*	miRNA-target	AP1/FUL, SOC1, AGL24	Key regulator in the age-dependent flowering pathway [31]
Trichome development	*AtSPL9*	miRNA-target; Protein–protein interaction	TCL1, TRY, TOE1	Suppresses trichome development [50,67]
Cuticular wax deposition	*AtSPL9*	miRNA-target; Protein–protein interaction	CER1	Environmental signal integrator for wax biosynthesis [68]

**Table 2:** Overview of SPL9 and its regulatory role in stress.

Stress	SPL9	Activity Regulation	Key Targets	Primary Function
Drought	*AtSPL9*	miRNA-target; Protein–protein interaction	DFR, PAP1; CER1, CER4	Divergent roles in anthocyanin-and wax-based drought resistance [34,103]
Heat	*AtSPL9*	miRNA-target	Auxin sensitivity-related genes	Regulating thermoresponsive hypocotyl elongation [104]
Cold	*AtSPL9*	Independent by miRNA-target	CBF2	Involved in age-dependent freezing tolerance [33]
	*IPA1*	Phosphorylated by OsSAPK6	OsCBF3	Play a role in chilling stress-tolerant rice breeding [105]
Disease	*AtSPL9*	miRNA-target	NA	Positively regulates ROS and SA signaling; negatively modulates JA signaling [35,36]
insect	*AtSPL9*	miRNA-target	JAZ	Core-regulator of age-JA crosstalk in insect defense [6]

## Data Availability

No new data were created or analyzed in this study.

## Supplementary Materials

The following supporting information can be downloaded at: https://www.mdpi.com/article/10.3390/plants15050814/s1, Table S1: Summary of SPL9 and its orthologs in different plant species.

## Abbreviations

An-1	The major QTL *Awn-1* that encodes a bHLH protein
AGL42	AGA-MOUS-LIKE 24
AP1/FUL	APETALA1/FRUITFULL
BOP1/2	BLADE-ON-PETIOLE 1 and 2
BRC1	BRANCHED1
CYCD3	CyclinD3
D53	DWARF 53
DEWAX	DECREASE WAX BIOSYNTHESIS
EGL3	ENHANCER OF GLABRA 3
FAR1	Far-Red Impaired Response 1
FHY3	FAR-RED ELONGATED HYPOCOTYL3
GIS	GLABROUS INFLORESCENCE STEMS
GL1/3	GLABRA 1 and 3
GmENOD40-1	Glycine max Early Nodulin 40-1
GmNINa	Glycine max nodule inception a (GmNINa)
IPA1	Ideal Plant Architecture 1
LFY	LEAFY
LOX2	Lipoxygenase 2
MYS1/2	MYB-SHAQKYF 1 and 2
PAP1	Production of anthocyanin pigment 1
PRC1/2	Polycomb Repressive Complex1 and 2
PKL-HDA9	PICKLE- histone deacetylase 9
SMXL6/7/8	SUPPRESSOR OF MORE AXILLARY GROWTH2-LIKE proteins
SMZ	SCHLAFMUTZE
SNZ	SCHNARCHZAPFEN
SnRK2.6	SNF1-related protein kinase 2.6
SOC1	SUPPRESSOR OF OVEREXPRESSION OF CO 1
SPL9	SQUAMOSA-PROMOTER BINDING PROTEIN LIKE 9
TCL1	TRICHOMELESS 1
TOE1	TARGET OF EAT1
TRY	TRIPTYCHON
TTG1	TRANSPARENT TESTA GLABRA 1
VSP2	Vegetative Storage Protein 2
